# Advancing interprofessional collaboration: exploring physicians’ perceptions, preferences, and barriers regarding pharmacists’ roles in Jordan

**DOI:** 10.1080/20523211.2026.2736421

**Published:** 2026-09-30

**Authors:** Zelal Kharaba, Karem H. Alzoubi, Mai Riziq, Lana Zuriegat, Rana Abu-Farha, Anan Jarab, Azza Ramadan, Feras Jirjees, Hala Afifi, Farah K. El-zubi, Yassen Alfoteih, Manal Buabeid

**Affiliations:** a Department of Pharmacy Practice and Pharmacotherapeutics, College of Pharmacy, University of Sharjah, Sharjah, United Arab Emirates; b Research Institute of Medical and Health Sciences, University of Sharjah, Sharjah, United Arab Emirates; c Faculty of Medical Sciences, Newcastle University, Newcastle upon Tyne, UK; d Department of Clinical Pharmacy, Faculty of Pharmacy, Jordan University of Science and Technology, Irbid, Jordan; e Faculty of Education, University of Ottawa, Ottawa, ON, Canada; f Clinical Pharmacy and Therapeutics Department, Faculty of Pharmacy, Applied Science Private University, Amman, Jordan; g Department of Pharmacology & Therapeutics, College of Medicine and Health Sciences, United Arab Emirates University, Al Ain, United Arab Emirates; h Department of Pharmaceutical Sciences, College of Pharmacy, Al Ain University, Abu Dhabi, United Arab Emirates; i AAU Health and Biomedical Research Center, Al Ain University, Abu Dhabi, United Arab Emirates; j Research Directorate City University Ajman, Ajman, United Arab Emirates; k Department of Internal Medicine, King Abdulla University Hospital, Ramtha, Jordan; l College of Dentistry, City University Ajman, Ajman, United Arab Emirates; m College of Humanities, City University Ajman, Ajman, United Arab Emirates; n Department of Pharmacy, Fatima College of Health Sciences, Abu Dhabi, United Arab Emirates

**Keywords:** Interprofessional, physician–pharmacist, physician perception, healthcare barriers, medication management

## Abstract

**Background:**

Interprofessional collaboration is crucial for improving healthcare delivery and addressing workforce challenges. Physician-pharmacist collaboration enhances patient care, improves medication management and health outcomes. While patients’ perspectives have been studied, physicians’ views in Jordan remain under-investigated. This study assessed physicians’ perceptions, preferences, and barriers to pharmacist integration.

**Methods:**

A cross-sectional survey was conducted among practicing physicians between April 1st and September 30th, 2025. Despite an extended six-month recruitment period, severe clinical time constraints resulted in a final sample of 297 participants, representing a 21.6% shortfall from the initial target of 379 and yielding a post-hoc margin of error of 5.66%. Using a convenience sampling approach with multi-channel recruitment, the survey was distributed via professional social media networks, direct electronic contacts, and targeted in-person clinic visits. Data were analysed using descriptive and inferential statistics to summarise the variables and examine the sample characteristics.

**Results:**

The majority of physicians (95.3%) had collaborated with pharmacists, with face-to-face communication being the most preferred method (72.7%). The responding physicians generally perceived positive roles for pharmacists in advising on drug interactions (60.3%), improving patient adherence (60.3%), and adjusting medication dosages (58.9%). A significant association was observed between practice sector and physicians’ perception scores (*p* = 0.021), with physicians working in the private health sector having lower perception scores towards pharmacists’ professional roles compared with those working in the governmental health sector.. Key barriers included the logistics of dealing with multiple healthcare professionals (54.9%), lack of face-to-face communication (52.2%), and fragmentation of care (49.2%). Financial constraints (46.5%) and concerns over shared patient responsibility (46.6%) were also significant barriers.

**Conclusion:**

Although Jordanian physicians reported positive perceptions of pharmacists’ expertise, systemic barriers and traditional role boundaries appear to hinder effective practice integration. Enhancing structured communication, implementing joint interprofessional training, and enacting target healthcare policy reforms are necessary to fostering effective physician-pharmacist collaboration and improving patient care outcomes.

## 1. Background

Interprofessional collaboration among healthcare providers is widely acknowledged as a crucial strategy for delivering comprehensive care, strengthening healthcare systems, and addressing the global health workforce crisis. According to the World Health Organization (WHO), interprofessional collaboration occurs when multiple healthcare workers from different professional backgrounds work together with patients, families, carers, and communities to deliver the highest quality of care, whereas collaborative practice is the structural mechanism through which this team-based care is actualised within the health services system (World Health Organization, 2010). Clarifying this distinction is essential, as the interactive process of interprofessional collaboration between pharmacists and physicians serves as the foundation for successful collaborative practice. Studies have demonstrated that effective interprofessional collaboration between pharmacists and physicians enhances patient care planning, documentation, and implementation (Kharaba et al., 2022a; Reeves et al., 2017).

Pharmacists are recognised as among the most accessible healthcare professionals who play essential roles in patient care within various community and healthcare settings. Furthermore, pharmacists’ interventions directly influence the health of patients and drive quality improvement in pharmaceutical services (Kharaba et al., 2026). Recognising these professional responsibilities is essential to fostering effective collaborative practice.

In Jordan, while early institutional frameworks initially defined the pharmacist's role around basic medication dispensing and patient counseling (Ayob et al., 2005), clinical pharmacy practice has expanded significantly over the past decade. Today, clinical pharmacists in Jordan are actively integrated into multidisciplinary hospital teams, performing advanced clinical duties such as ward-round participation, comprehensive medication reconciliation, active management of drug-related problems, and collaborative chronic disease management (Al-azzam et al., 2013; Ayob et al., 2005). Effective interprofessional collaboration is defined by a focus on patient-centred care, shared objectives, complementary skills, negotiation, joint decision-making, and mutual respect (Albassam et al., 2020). Several factors influence collaboration, including individual demographics and beliefs, aspects of professional practice, and critical elements like trust and communication (Doucette et al., 2005). Consistently, literature shows that physicians acknowledge the vital role pharmacists play in managing drug side effects, adjusting dosages, advising on drug interactions, and counseling patients (Albassam et al., 2020). These efforts collectively enhance health outcomes and reduce hospitalisations (Foot et al., 2022; Matzke et al., 2018). Despite these benefits, challenges to effective collaboration persist, including physical separation, limited access to medical records, insufficient interprofessional training, time constraints, and significant communication barriers that hinder optimal patient-pharmacist interaction (Guthrie et al., 2017; Kharaba et al., 2025). Published baselines in Jordan have examined historical physicians’ perceptions of pharmacists’ roles, revealing traditional hesitations regarding independent pharmacist prescribing alongside strong support for educational roles (AbuRuz et al., 2012). However, regional interprofessional dynamics have accelerated rapidly. As a prominent post-pandemic regional example, a 2024 study by Alssageer highlighted substantial hurdles to interprofessional collaboration, revealing that 61.8% of physicians felt unable to communicate effectively with clinical pharmacists, while 43.6% recognised deep-seated traditional opposition between the two professions as a primary barrier (Alssageer et al., 2024). Furthermore, the role of pharmacists in providing pharmaceutical care has evolved since the COVID-19 pandemic (Kharaba et al., 2022b), with greater involvement in medication dispensing, telepharmacy, and patient-centred services (Unni et al., 2021). This shift may have altered physicians’ perceptions of these expanded roles of pharmacists. Despite these evolving dynamics, existing research has yet to fully address how these shifts impact day-to-day collaboration with physicians, particularly regarding the systemic barriers and preferred communication channels of practicing physicians (Tahaineh et al., 2009). Understanding these specific operational factors is essential to move past general attitude assessments and design practical, integrated care models. This study addresses this gap by providing a contemporary, post-pandemic evaluation of physicians’ perceptions, the direct obstacles they encounter, and their preferred pathways for structured collaboration in Jordan. Thus, this study aimed to assess physicians’ perceptions, identify their preferences, and evaluate the barriers they encounter regarding interprofessional collaboration with pharmacists in Jordan.

## 2. Methods

### 2.1. Study design, sample, and setting

A cross-sectional study was designed and reported in accordance with the STROBE guidelines for observational studies. The study was conducted between the April 1st, and September 30th, 2025, and targeted physicians registered with the Jordan Medical Association, including those practicing in hospitals and clinics, in public and private sectors. The inclusion criteria allowed participation from all actively practicing physicians in Jordan, with the exclusion of those not currently engaged in clinical practice. To ensure a geographically diverse sample, the survey was distributed across major cities and regions in Jordan, capturing a broad representation of physicians nationwide.

### 2.2. Data collection

To minimise selection bias and maximise outreach within a busy clinical population, a convenience sampling approach with multi-channel recruitment was employed. The research team initially distributed the standardised electronic survey link via professional social media networks (such as Facebook, WhatsApp, and Instagram) and direct electronic contacts to a targeted cohort of 550 practicing physicians. To address the low response rates typical of practicing clinicians and optimise data collection, research assistants also conducted targeted, in-person clinic visits. During these visits, physicians who met the strict inclusion and exclusion criteria were invited to complete the anonymous survey on-site. Regular bi-weekly digital reminders were also utilised throughout the data collection period to enhance the response rate.

An introductory paragraph in the questionnaire clearly outlined the eligibility criteria, ensuring that only qualified physicians participated. To minimise response bias, the questionnaire was completed anonymously, and participants were assured that their responses would remain confidential.

### 2.3. Study questionnaires and validation

A comprehensive literature review was conducted to identify existing studies on interprofessional collaboration between physicians and pharmacists related to the study aims. The survey instrument was adapted from validated questionnaires previously used in studies conducted in Canada and Kuwait (Albassam et al., 2020; Kelly et al., 2013). To ensure structural, cultural, and contextual suitability for the Jordanian clinical environment, a comprehensive adaptation and qualitative validation of the instrument was conducted. First, an expert panel of four specialists – comprising two senior academic pharmacy researchers and two practicing clinical pharmacists affiliated with the University of Sharjah and Jordan University of Science and Technology – systematically reviewed the items adapted from the Canadian and Kuwaiti tools. The adaptation process specifically focused on realigning institutional healthcare classifications (primary, secondary, and tertiary settings) and professional communication channels to accurately reflect the distinct administrative frameworks and workflow realities of the Jordanian healthcare system. Furthermore, while the questionnaire was kept in English – the standard medium of professional instruction and practice for Jordanian physicians – the expert panel adjusted the phrasing of operational barrier items to ensure situational relevance and eliminate regional ambiguity. Following these qualitative modifications, the adapted tool was subjected to a pilot test among sixteen practicing physicians to confirm optimal face validity and readability prior to formal distribution.

To prevent clinical ambiguity, the survey explicitly defined the distinct roles evaluated: community pharmacists were defined as those operating within retail, primary care community settings, while clinical pharmacists were defined as hospital-based practitioners integrated into multidisciplinary ward teams. Where a specific setting was not indicated, the general term ‘pharmacist’ was used to capture physicians’ overarching perceptions of the profession across the broader healthcare system. The final questionnaire consisted of six sections, each containing close-ended questions. The first section collected demographic information, including age, sex, years of practice, geographic location, workplace setting (hospital and clinic), and sector (public or private). The second section included four items assessing physicians’ attitudes towards collaborative practice. The third section comprised eight questions exploring physicians’ perceptions of pharmacists’ professional roles. The fourth section contained six items evaluating preferred methods of communication for collaboration. The fifth section included seven questions identifying potential areas for enhanced collaboration. The final section featured nine questions addressing perceived barriers to collaboration. Responses were recorded on a 5-point Likert scale, with 1 indicating strong disagreement and 5 indicating strong agreement. For the final descriptive analysis, these responses were grouped into three primary categories: agreement (strongly agree/agree), neutral, and disagreement (strongly disagree/disagree).

A composite perception score was calculated by summing the responses to the eight items assessing physicians’ perceptions of pharmacists’ professional roles. Each item was scored on a 5-point Likert scale, with responses ranging from 1 (strongly disagree) to 5 (strongly agree), resulting in a total possible score ranging from 8 to 40, where higher scores indicated more positive perceptions.

To ensure face validity, clarity, and readability, a single pilot study involving sixteen practicing physicians was conducted. These participants evaluated the questionnaire’s format, length, comprehension, and overall impression. Minor revisions were made based on their feedback to optimise the final instrument. To maintain data integrity, all sixteen physicians who participated in this validation and pilot stage were strictly excluded from the final study sample.

### 2.4. Sample size calculation

As of January 2021, the number of registered physicians in Jordan was approximately 28,000 (Alhamad et al., 2021). Based on the Raosoft® sample size calculator (Raosoft, 2026) a minimum of 379 participants was estimated for a 5% margin of error at a 95% confidence level. The final sample comprised 297 participants, representing a 21.6% shortfall due to low digital response rates. To overcome this, the team conducted targeted in-person clinic visits over a six-month period. To prevent response or interviewer bias, all data collection assistants received strict orientation and standardisation training led by the Principal Investigator (ZK) to act strictly as neutral facilitators, distributing and collecting the anonymous, self-administered surveys without influencing participants’ selections. This final sample size yields a post-hoc margin of error of 5.66%, which remains statistically robust for exploratory clinician surveys (Dillman et al., 2014).

### 2.5. Ethical considerations

Ethical approval for the study was obtained from the Institutional Review Board (IRB) at Applied Science Private University, Amman, Jordan under protocol number [2025-PHA-9] on 17 March 2025 following an expedited review procedure. Electronic informed consent was obtained from all participating physicians prior to their involvement in the study. The study adhered to the ethical principles outlined in the Declaration of Helsinki, ensuring the protection of participants’ rights and confidentiality throughout the research process.

### 2.6. Statistical analysis

Data analysis was performed using the Statistical Package for the Social Sciences (SPSS) version 29 (SPSS Inc., Chicago, IL, USA). Descriptive statistics were used to summarise the study variables. Categorical variables were presented as frequencies and percentages, while continuous variables were reported as medians with interquartile ranges (IQR). The Shapiro–Wilk test was used to assess the normality of continuous data. Univariate and multivariate linear regression analyses were performed to examine the association between physicians’ perception scores and each independent variable. Regression coefficients (B) with 95% confidence intervals (CIs) were reported. Model assumptions, including normality of residuals, linearity, homoscedasticity, and multicollinearity, were assessed before interpreting the final model. A *p*-value < 0.05 was considered statistically significant for all statistical tests.

## 3. Results

A total of 550 physicians were invited to participate in the survey after consent, of whom 330 completed the questionnaire (response rate 60%). Questionnaires were excluded from the final analysis only if entire major evaluation sections (such as the complete perception or barrier scales) were left blank. Based on this objective rule, 33 questionnaires were excluded due to completely missing sections, leaving a final sample of 297 questionnaires for the analysis. The median age was 29 years (IQR = 5). More details of the sociodemographic are presented in Table 1.

**Table 1.** Sociodemographic characteristics of the participating physicians (n = 297).

Parameter	Median (IQR)	Frequency (%)
Age	29.0 (IQR = 5.0)	
Sex		
Male		237 (79.8%)
Female		60 (20.2%)
Years of professional experience	4.0 (IQR 4.0)	
Working province		
Center of Jordan (Amman, Zarqa, Balqa, and Madaba)		179 (60.3%)
North of Jordan (Irbid, Ajloun, Jerash, and Mafraq)		99 (33.3%)
South of Jordan (Karak, Maan, Tafila, and Aqaba)		19 (6.4%)
Healthcare system		
Primary sector		206 (69.4%)
Secondary sector		47 (15.8%)
Tertiary sector		44 (14.8%)
Working sector		
Governmental health sector		147 (49.5%)
Private health sector		150 (50.5%)
Collaborated with pharmacists during professional practice		
Yes		283 (95.3%)
No		14 (4.7%)

IQR: Interquartile range.

The participants reported several methods of communication with pharmacists, Figure 1. Face-to-face communication was the preferred method (72.7%), followed by telephone communication (60.9%) and paper communication (43.8%).

**Figure 1.**
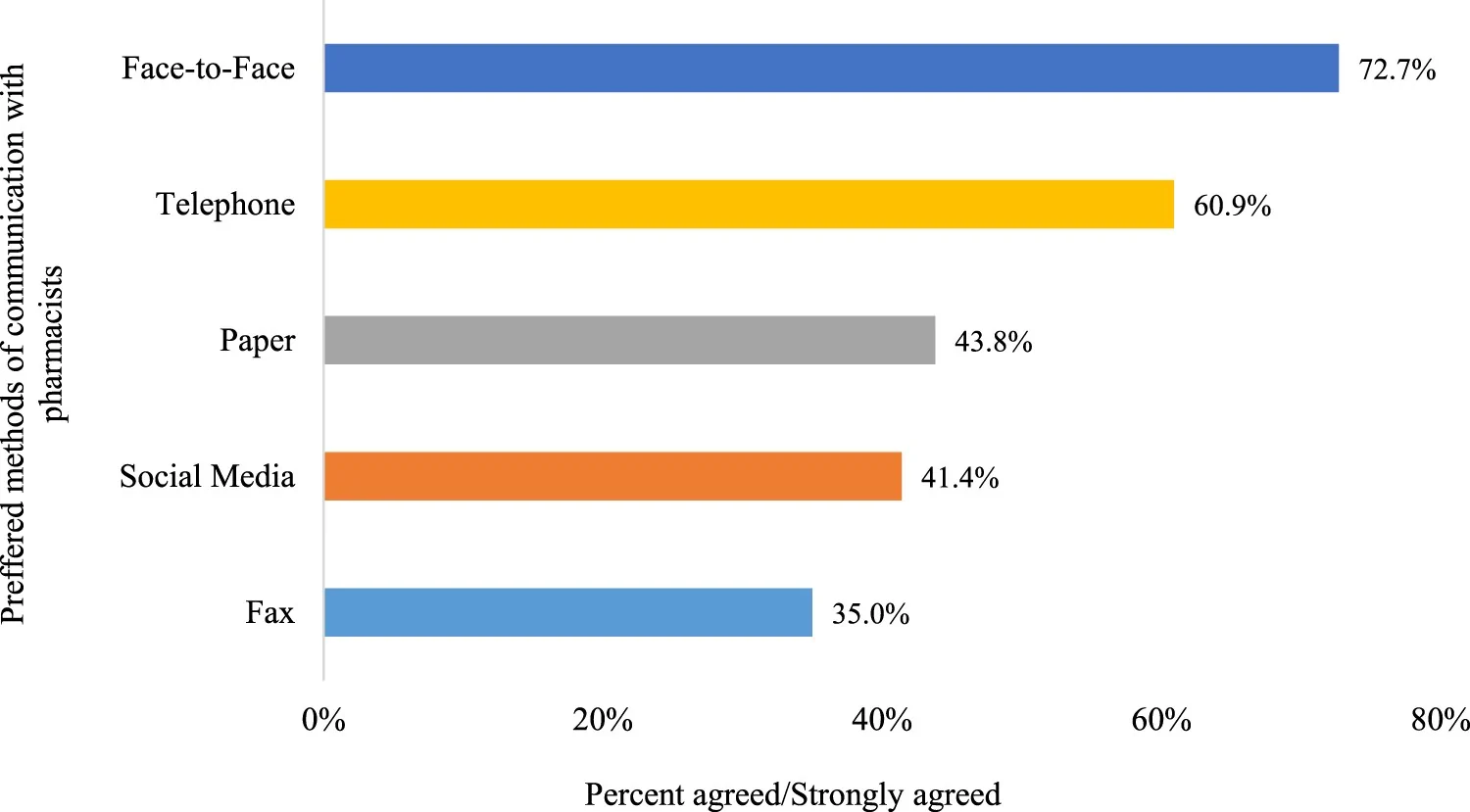
Preferred methods of communication with pharmacists among participating physicians (n = 297). Values represent the percentage of physicians who selected each communication method as preferred.

Figure 2 shows physicians’ perceptions towards the professional role of pharmacists in improving patient care. The most agreed-upon roles were pharmacists’ involvement in advising about drug interactions and improving patient adherence. Other commonly recognised roles included assisting with medication dose adjustment and providing drug information to support patient-specific therapeutic decision-making.

**Figure 2.**
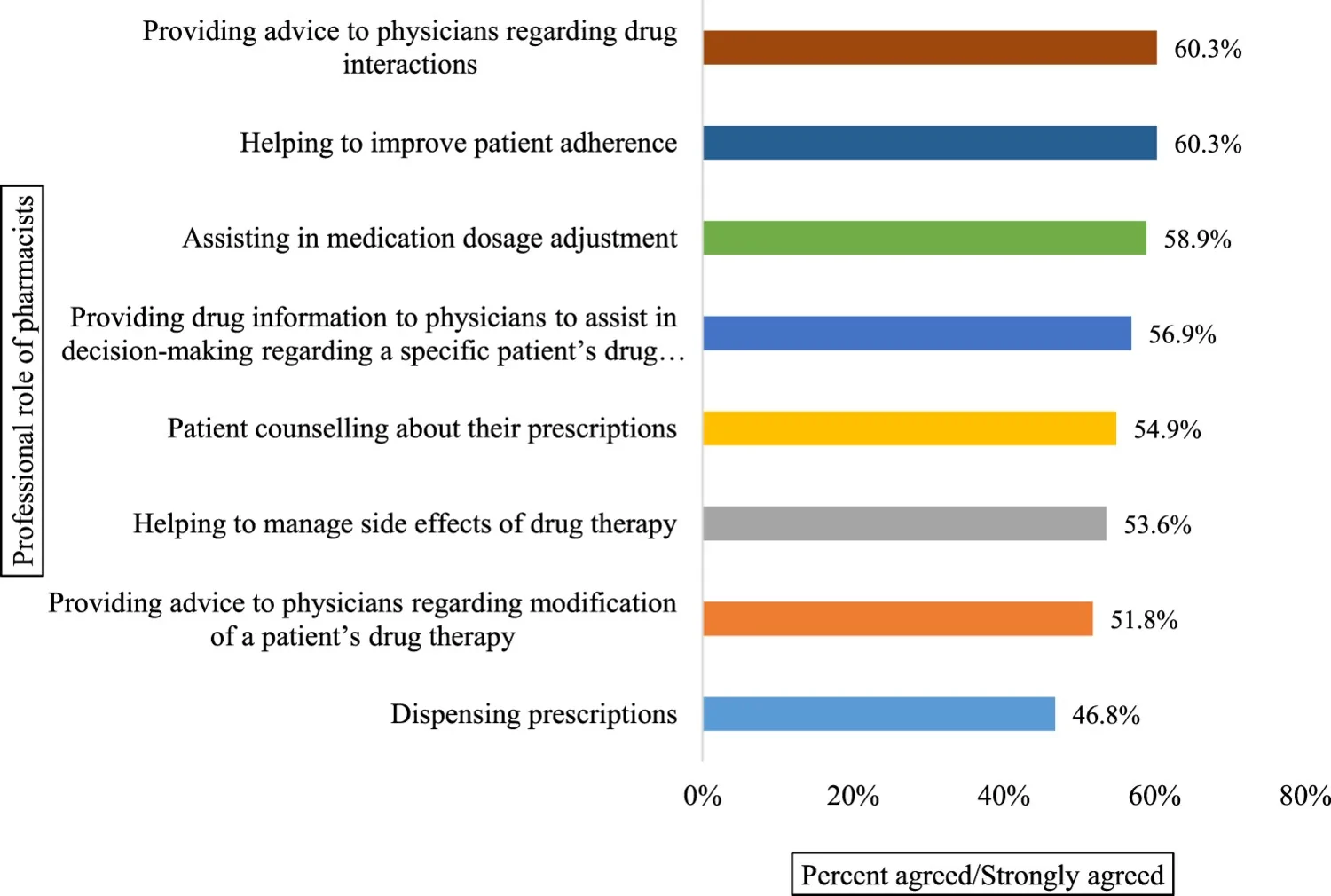
Physicians’ perceptions of pharmacists’ professional roles in improving patient care (n = 297). Values represent the percentage of physicians who agreed or strongly agreed that pharmacists have each specified role.

The participating physicians were asked to identify areas where further collaboration with pharmacists is needed in the future, Table 2. The results showed that the main area was providing advice about drug interactions (64.6%), followed by improving patient adherence (62.6%), and assisting in medication selection (61.9%).

**Table 2.** Physicians’ perceptions towards areas that need further collaboration between physicians and pharmacists (n = 297).

Future collaboration with pharmacists	Strongly disagreed	Disagreed	Neutral	Agreed	Strongly agreed
	No. (%)				
Patient counselling	26 (8.8)	26 (8.8)	98 (33.0)	69 (23.2)	78 (26.3)
Helping in the management of side effects of drug	16 (5.4)	28 (9.4)	78 (26.3)	83 (27.9)	92 (31.0)
Making recommendations to modify a patient’s drug therapy	19 (6.4)	24 (8.1)	83 (27.9)	92 (31.0)	79 (26.6)
Assisting in medication dosage adjustment	23 (7.7)	27 (9.1)	78 (26.3)	85 (28.6)	84 (28.3)
Providing drug information to help select a medication	18 (6.1)	22 (7.4)	73 (24.6)	82 (27.6)	102 (34.3)
Providing advice regarding drug interactions	11 (3.7)	23 (7.7)	71 (23.9)	83 (27.9)	109 (36.7)
Helping to improve patient adherence	13 (4.4)	25 (8.4)	73 (24.6)	88 (29.6)	98 (33.0)

Table 3 summarises the barriers perceived by physicians that hinder effective collaboration with pharmacists. The most commonly reported barriers were the need to deal with multiple healthcare professionals (54.9%) and lack of face-to-face communication (52.2%). Other barriers reported by physicians included fragmentation of care due to involvement of multiple healthcare providers, liability concerns, lack of financial compensation, and limited time availability.

**Table 3.** Barriers perceived by physicians towards collaboration with pharmacists (n = 297).

Future collaboration with pharmacists	Strongly disagreed	Disagreed	Neutral	Agreed	Strongly agreed
	No. (%)				
Lack of time	31 (10.4)	41 (13.8)	104 (35.0)	64 (21.5)	57 (19.2)
Lack of face-to-face communication	28 (9.4)	34 (11.4)	80 (26.9)	76 (25.6)	79 (26.6)
Involvement of multiple healthcare providers resulting in fragmentation of care	15 (5.1)	25 (8.4)	111 (37.4)	95 (32.0)	51 (17.2)
Lack of financial compensation	24 (8.1)	36 (12.1)	99 (33.3)	73 (24.6)	65 (21.9)
Liability over shared patient information	21 (7.1)	40 (13.5)	111 (37.4)	82 (27.6)	43 (14.5)
Liability over shared patient responsibility	18 (6.1)	34 (11.4)	106 (35.7)	85 (28.6)	54 (18.2)
Need to deal with multiple healthcare professionals	19 (6.4)	22 (7.4)	93 (31.3)	92 (31.0)	71 (23.9)
Lack of belief that collaborative practice will improve patient care	41 (13.8)	37 (12.5)	101 (34.0)	58 (19.5)	60 (20.2)
Lack of confidence in the pharmacist’s knowledge or skills to take their advice in patient care issues	36 (12.1)	47 (15.8)	101 (34.0)	58 (19.5)	55 (18.5)

Table 4 presents the factors associated with physicians’ perceptions of pharmacists’ professional roles in improving patient care. In the simple linear regression analysis, working in the private health sector was significantly associated with lower perception scores compared with the governmental health sector (B = −2.253, 95% CI: −4.117 to −0.390, *p* = 0.018). This association remained significant after adjusting for age, sex, years of professional experience, working province, healthcare system, and previous collaboration with pharmacists (adjusted B = −2.366, 95% CI: −4.370 to −0.362, *p* = 0.021). The multiple linear regression model explained 3.7% of the variance in physicians’ perception scores (R² = 0.037).

**Table 4.** Factors associated with physicians’ perceptions of pharmacists’ professional roles in improving patient care (n = 297).

Parameter	Perception score towards pharmacists’ professional roles					
	Beta	95% CI of Beta	*P*-value#	Beta	95% CI of Beta	*P*-value$
Age	0.056	−0.124 to 0.236	0.543	−0.106	−0.473 to 0.261	0.569
Gender						
Male	Reference					
Female	0.770	−1.571 to 3.112	0.518	0.904	−1.485 to 3.293	0.457
Professional experience	0.084	−0.129 to 0.298	0.439	0.142	−0.293 to 0.577	0.522
Location						
Center of Jordan	Reference					
North of Jordan	0.924	−1.069 to 2.917	0.362	0.993	−1.077 to 3.064	0.346
South of Jordan	0.440	−3.304 to 4.284	0.822	0.488	−3.555 to 4.531	0.812
Working sector						
Governmental health sector	Reference					
Private health sector	−2.253	−4.117 to −0.390	0.018	−2.366	−4.370 to −0.362	0.021*
Healthcare system						
Primary sector	Reference					
Secondary sector	−1.394	−3.967 to 1.178	0.287	−0.766	−3.464 to 1.932	0.577
Tertiary sector	1.497	−1.164 to 4.122	0.272	0.903	−2.001 to 3.806	0.541
Previous collaboration with pharmacists						
No	Reference					
Yes	−2.587	−7.016 to 18.43	0.251	−2.986	−7.640 to 1.667	0.208

B: Unstandardised regression coefficient. # Obtained using simple linear regression. $ Obtained using multiple linear regression. * Statistically significant at *p* < 0.05.

## 4. Discussion

This study provides valuable insights into physicians’ perceptions, preferences, as well as barriers that face, regarding interprofessional collaboration with pharmacists in Jordan. The findings highlight that the majority of participants have collaborated with pharmacists at some point in their practice; however the study also identifies several factors that affect the effectiveness and extent of this collaboration.

Physicians’ perceptions of the role of pharmacists probably play a vital role in the establishment of collaborative practice in the healthcare system (Alkhateeb et al., 2009). The majority of physicians in our study recognised the value of pharmacists in improving patient care, particularly in areas related to drug interactions, patient adherence, and medication dose adjustments. These results suggest a growing recognition among physicians of pharmacists’ expertise in medication management and align with previous research findings (Dubán et al., 2017; Kucukarslan et al., 2011). However, physicians in our study were less likely to view pharmacists as key contributors to medication dispensing. We hypothesise that this finding reflects deeply ingrained, traditional professional boundaries within the healthcare system, where clinical diagnosing and prescribing are viewed as the physician's domain, while dispensing is treated as an independent, technical duty of the pharmacist (Nazer & Tuffaha, 2017). Because dispensing is historically viewed as a standalone, non-collaborative task falling strictly within the pharmacist's autonomous scope of practice, physicians may not perceive it as an activity requiring active interprofessional collaboration. This division of labour is well-documented in regional literature, where physicians support pharmacists’ clinical counseling roles but remain hesitant about integrating collaborative protocols into traditional technical tasks (Nazer & Tuffaha, 2017). Further qualitative research is required to explore how physicians define the boundaries between independent tasks and collaborative practices.

While understanding these clinician attitudes provides an important baseline, the practical value of physician-pharmacist collaboration is best illustrated by its measurable impact in real-world clinical models. Transitioning from positive perceptions to actual practice relies heavily on integrating structured clinical pharmacy services into multidisciplinary teams. For instance, a recent two-year evaluation of a clinical pharmacy ambulatory model in an Italian tertiary hospital demonstrated that embedding pharmacist-led services – such as medication reviews and patient education – generated therapeutic recommendations with a physician acceptance rate exceeding 70% (Mengato et al., 2025). Similarly, a national seamless care initiative in Slovenia successfully implemented a fully reimbursed, protocol-driven medication reconciliation programme across hospitals and community pharmacies, which significantly reduced medication-related errors during care transitions (Stuhec, 2025). These established international models show that the clinical expectations and collaborative willingness voiced by physicians can translate into functional, high-impact partnerships when supported by clear institutional frameworks and national reimbursement protocols (The Interprofessional Education Collaborative, 2023).

Communication between physicians and pharmacists is crucial for effective collaboration. Face-to-face interaction emerged as the preferred mode, followed by telephone communication and written communication via paper. These findings are consistent with those reported in similar studies from Kuwait, Slovakia and Iran (Albassam et al., 2020; Dubán et al., 2017; Hashemian et al., 2016). The preference for direct communication underscores the importance of real-time, interactive discussions in clinical decision-making. The relatively low preference for social media and fax suggests concerns over privacy, security, and the reliability of these communication methods. This preference for face-to-face interaction underscores the need to enhance structured communication channels in clinical settings.

Our descriptive findings clearly show that physicians in Jordan prefer immediate, synchronous communication channels – specifically face-to-face (72.7%) and telephone contact (60.9%) – over asynchronous digital communication. This preference suggests that clinicians prioritise real-time dialogue to facilitate rapid decision-making in busy practice settings. Consequently, traditional paper-based methods (43.8%) continue to serve as the primary, albeit fragmented, medium for documented interprofessional collaboration in local healthcare settings.

Physicians demonstrated a strong interest in enhancing their collaboration with pharmacists, particularly in areas such as providing drug interaction advice, improving patient adherence, and assisting with medication selection. These findings align with previous research (Albassam et al., 2020; Dubán et al., 2017), highlighting an appreciation for pharmacists’ expertise in pharmacotherapeutics and their role in optimising medication regimens. The extent to which physicians value specific pharmacist activities and perceive their competence in these functions has influenced physician attitudes and collaboration (Ranelli & Biss, 2000). Kucukarslan et al. (2011) suggested that physicians’ belief in the potential for collaboration to enhance medication adherence may significantly impact their willingness to work with community pharmacists (Kucukarslan et al., 2011).

Despite the positive perceptions of pharmacists’ roles, several barriers were identified that hinder effective collaboration. The most significant barrier was the need to deal with multiple healthcare professionals, followed by a lack of face-to-face communication and fragmentation of care due to multiple providers. These findings indicate that system-related factors, including healthcare structure and interprofessional coordination, are pivotal in shaping the success of collaborative efforts. They emphasise the necessity of implementing interventional strategies to address these barriers, facilitating the integration of collaborative practice in primary healthcare settings in Jordan. Lack of face-to-face communication was reported as a significant barrier to collaborative practice. These findings highlight the importance of fostering direct communication between physicians and pharmacists, as it has been shown to positively influence the development of interprofessional relationships (Doucette et al., 2005). Strategies should be developed to enhance communication skills and create an environment that encourages frequent and comfortable interactions between both professions. Face-to-face communication can be facilitated through joint continuing education programmes and professional events. Hashemian et al. (2016) recommended the use of cost-effective, time-efficient applications with video call capabilities to support face-to-face discussions on medication-related issues. Additionally, the potential fragmentation of patient care due to the involvement of multiple healthcare professionals can be addressed through coordinated care and clear role specification (Hashemian et al., 2016). Establishing shared expectations for each professional’s role in patient care ensures a complementary approach, where each practitioner acknowledges and values the other’s contributions. Role specification has been identified as a key factor in the success of collaborative practices, providing a structured framework for effective interactions (Al-Jumaili et al., 2017). Additionally, it may help mitigate time constraints, which respondents highlighted as a barrier to collaboration. Further qualitative research is needed to gain deeper insights into the barriers and facilitators of collaboration, supporting the adoption and implementation of a consistent, evidence-based, and integrated physician-pharmacist partnership in Jordan.

Financial constraints were identified as a significant barrier by nearly half of the participants (46.5%), a finding that carries profound policy implications for Jordan's mixed healthcare system. This barrier is deeply tied to the structural differences between Jordan's public and private sectors. In the private sector, which operates largely on a fee-for-service model, physicians face a clear financial disincentive to collaborate because current insurance structures do not provide reimbursement codes for interprofessional consultations, joint case reviews, or comprehensive medication therapy management (MTM). Conversely, in the public sector, where physicians are salaried, these financial constraints are felt through institutional underfunding, which restricts the hiring and placement of dedicated clinical pharmacists in busy hospital wards. To bridge this gap, Jordanian health policymakers must transition from purely encouraging collaboration to actively incentivising it. Specifically, the Ministry of Health, in coordination with private health insurance companies, should introduce dedicated clinical billing codes that reimburse physicians for time spent in documented, collaborative clinical reviews with pharmacists. Furthermore, allocating dedicated public health budgets to expand clinical pharmacy departments in governmental hospitals is essential. Without structural reforms to billing and reimbursement models, physicians’ positive attitudes towards pharmacists’ expertise will continue to be bottlenecked by financial and operational realities.

Factors associated with physicians’ perceptions of pharmacists’ professional roles were explored using multiple linear regression analysis. No significant associations were observed between perception scores and age, sex, years of professional experience, working province, healthcare system, or previous collaboration with pharmacists. However, physicians working in the private health sector had significantly lower perception scores compared with those working in the governmental sector. In line with our regression model, no statistically significant differences in perception scores were observed based on the sex of the participating physicians.. Factors associated with physicians’ perceptions of pharmacists’ professional roles were explored using multiple linear regression analysis. No significant associations were observed between perception scores and demographic variables such as age, sex, or years of professional experience. This lack of statistical significance across baseline demographics suggests that clinical perceptions are relatively uniform across these personal characteristics within our sample population, highlighting that structural workplace factors play a more defining role than individual traits.

Finally, a significant association was observed between the working sector and collaboration (*p* = 0.031), with governmental sector physicians being more likely to collaborate with pharmacists than those in the private sector. Although our study did not evaluate organisational policies, we hypothesise that this variation might be related to structural differences, such as more standardised institutional protocols or integrated team environments within public facilities compared to private practices (Nazer & Tuffaha, 2017). Further investigation is required to determine whether these organisational factors explain the differences in collaboration rates between the sectors.

### 4.1. Strengths and limitations

This study offers a comprehensive look at how physicians in Jordan view and experience collaboration with pharmacists, using a geographically diverse sample and validated methods. A key strength is our adherence to the STROBE reporting guidelines, ensuring methodological transparency and completeness. However, several limitations must be considered.

First, our cross-sectional design prevents us from drawing causal conclusions, and relying on self-reported survey data can introduce response bias. Because participation was voluntary and anonymous, we faced a moderate, positive selection bias: physicians with positive views towards pharmacists or those who are highly connected online were likely more motivated to respond. Additionally, in-person recruiting naturally favoured busy, urban hospital hubs over remote or restricted private clinics. Coupled with a 40% non-response rate, these factors mean our findings likely overestimate positive perceptions and underestimate daily collaboration barriers. We mitigated this by using a dual-channel recruitment strategy (combining online outreach with intensive clinic visits), which successfully brought in a diverse mix of practice sectors and experience levels.

Second, our measurement tools and scope introduced some specific constraints:

- Scale Reliability: While our survey was based on validated tools and checked by an expert panel, the heavy use of categorical checkboxes meant we did not calculate internal consistency metrics (such as Cronbach's alpha) for the multi-item composite perception scale.
- Setting Ambiguity: We did not explicitly separate hospital-based clinical pharmacists from community pharmacists in the questionnaire. Since physician expectations vary widely depending on the practice setting, grouping them under a general category may have blurred some setting-specific nuances.
- One-Sided Perspective: This study focused exclusively on physicians. Because collaboration is bidirectional, the absence of matching data from pharmacists limits our ability to identify gaps or disagreements in how both professions experience daily teamwork.

Finally, our final sample of 297 physicians fell short of our original 379 target due to intense clinical workloads during the six-month study period. This 21.6% recruitment deficit increased our post-hoc margin of error to 5.66%, slightly exceeding the standard 5.0% threshold. This loss of precision means our findings cannot be generalised to the entire physician population of Jordan with absolute confidence.

Despite these limitations, this study serves as a highly valuable, realistic exploratory baseline. Our findings highlight critical areas for intervention and provide a foundation for future national studies using stratified, random sampling to capture both sides of the interprofessional relationship.

### 4.2. Implications for policy and practice

Our findings underscore the need for targeted, context-specific policy reforms, requiring coordinated actions from key healthcare stakeholders in Jordan to bridge the gap between positive clinician perceptions and systemic operational barriers. To translate these insights into practice, we propose a tripartite stakeholder framework:

- The Ministry of Health (MOH): The MOH should structurally update Hakeem – Jordan's national electronic health record system – to include dedicated, secure interprofessional communication portals. This integration would allow both hospital and community pharmacists to safely log clinical recommendations and coordinate directly with prescribing physicians in real time, bypassing traditional communication barriers. Additionally, to cement cooperative habits early in clinical careers, the MOH should mandate standardised interprofessional training modules for all healthcare professionals during their first two years of practice.
- Professional Associations and Regulatory Bodies (JPA, JMA, and JFDA): The Jordan Pharmacists Association (JPA) and the Jordan Medical Association (JMA), in coordination with the Jordan Food and Drug Administration (JFDA), must jointly develop and formally endorse standardised Collaborative Practice Agreements (CPAs). These legal frameworks should clearly define independent versus shared clinical boundaries in chronic disease management, dosage titration, and medication reconciliation, directly resolving physicians’ liability concerns over shared patient responsibility.
- Academic Institutions and CPD Providers: Jordan's leading academic institutions – such as the Jordan University of Science and Technology (JUST) and the University of Jordan (UJ) – should integrate mandatory, credit-bearing Interprofessional Education (IPE) modules into core medical and pharmacy curricula to dismantle professional silos early. Furthermore, the JMA and JPA should co-design accredited, joint continuing professional development (CPD) workshops to address the lack of face-to-face communication, helping practicing clinicians build mutual trust in daily practice.

### 4.3. Conclusion

This study highlights the growing recognition among physicians in Jordan of the valuable role pharmacists play in medication management, particularly in areas such as drug interactions, patient adherence, and dosage adjustments. However, systemic barriers, including fragmented care, lack of face-to-face communication, and financial constraints, hinder effective collaboration between the physicians and pharmacists. Future research should incorporate pharmacists’ perspectives and explore qualitative insights to further understand and address the complexities of interprofessional collaboration.

## Supplementary Material

STROBE checklist v4 combined PlosMedicine.docx

Revised STROBE 17July.docx

## Data Availability

The data sets generated and analysed during the current study are available from the corresponding author upon reasonable request.

## Data access statement

The authors had full access to all study data and are responsible for the integrity of the data and accuracy of the analysis.

## Use of generative artificial intelligence in scientific writing

During the preparation of this manuscript, the authors utilised the generative artificial intelligence platform Gemini (May 2026 version; Google LLC, Mountain View, CA, USA) to assist with grammatical polishing, refining sentence structure, and ensuring compliance with STROBE reporting guidelines. The AI tool was strictly used to improve readability and formatting; it was not used to create, analyse, or interpret any research data, nor did it influence the clinical findings or conclusions of the study. The authors maintain full accountability for the content and integrity of the final manuscript.
