# Supplementary material for: Advancing interprofessional collaboration: exploring physicians’ perceptions, preferences, and barriers regarding pharmacists’ roles in Jordan
Source: J Pharm Policy Pract. 2026 Sep 30;19(1):2736421. doi: 10.1080/20523211.2026.2736421 (PMC13629796; doi:10.1080/20523211.2026.2736421)
Supplement: Supplementary file 1 [file JPPP_A_2736421_SM5450.docx]

**STROBE Statement—Checklist of items that should be included in reports of cross-sectional studies**

| **Section / Topic** | **Item No.** | **Recommendation** | **Page No.** | **Relevant text and justifications from manuscript** |
| --- | --- | --- | --- | --- |
| **TITLE and ABSTRACT** | **1** | (a) Indicate the study’s design with a commonly used term in the title or the abstract | **1, 2** | *Title:* "...Exploring Physicians' Perceptions, Preferences, and Barriers Regarding Pharmacists' Roles in Jordan"    *Abstract:* "Methods: A cross-sectional survey was conducted..." |
|  |  | (b) Provide in the abstract an informative and balanced summary of what was done and what was found | **2** | Includes Background, Methods (n=297, 5.66% margin of error), Results (regression, barriers), and Conclusion. |
| **INTRODUCTION** |  |  |  |  |
| **Background / rationale** | **2** | Explain the scientific background and rationale for the investigation being reported | **3, 4** | "Interprofessional collaboration among healthcare providers is widely acknowledged... In Jordan, while early institutional frameworks... Today, clinical pharmacy practice has expanded..." |
| **Objectives** | **3** | State specific objectives, including any prespecified hypotheses | **4** | "Thus, this study aimed to assess physicians' perceptions, identify their preferences, and evaluate the barriers they encounter regarding interprofessional collaboration with pharmacists in Jordan." |
| **METHODS** |  |  |  |  |
| **Study design** | **4** | Present key elements of study design early in the paper | **5** | "A cross-sectional study was designed and reported in accordance with the STROBE guidelines for observational studies." |
| **Setting** | **5** | Describe the setting, locations, and relevant dates, including periods of recruitment, exposure, follow-up, and data collection | **5** | "The study was conducted between the April 1st, and September 30th , 2025, and targeted physicians registered with the Jordan Medical Association..." |
| **Participants** | **6** | (a) Give the eligibility criteria, and the sources and methods of selection of participants | **5** | "The inclusion criteria allowed participation from all actively practicing physicians in Jordan, with the exclusion of those not currently engaged in clinical practice." |
|  |  | (b) For matched studies, give matching criteria and number of exposed and unexposed | **N/A** | *Justification:* Not applicable as no matching criteria or matched cohorts were used in this descriptive cross-sectional design. |
| **Variables** | **7** | Clearly define all outcomes, exposures, predictors, potential confounders, and effect modifiers. Give diagnostic criteria, if applicable | **6, 7** | Primary outcomes: perception score (8–40 Likert scale), communication preferences, and structural barrier items. Predictors: age, sex, years of experience, setting, sector, and previous collaboration. |
| **Data sources / measurement** | **8*** | For each variable of interest, give sources of data and details of methods of assessment (measurement). Describe comparability of assessment methods if there is more than one group | **6** | "The survey instrument was adapted from validated questionnaires previously used in studies conducted in Canada and Kuwait..." |
| **Bias** | **9** | Describe any efforts to address potential sources of bias | **5, 7** | "...all data collection assistants received strict orientation and standardization training led by the Principal Investigator (ZK) to act strictly as neutral facilitators..." |
| **Study size** | **10** | Explain how the study size was arrived at | **7** | Target of 379 estimated using Raosoft (95% CI, 5% MoE). Achieved 297, yielding a highly robust 5.66% post-hoc margin of error. |
| **Quantitative variables** | **11** | Explain how quantitative variables were handled in the analyses. If applicable, describe which groupings were chosen and why | **7** | Continuous variables (age, experience) were kept continuous. Composite perception scores were calculated as sum scores (range 8–40). |
| **Statistical methods** | **12** | (a) Describe all statistical methods, including those used to control for confounding | **7, 8** | Descriptive stats (medians/IQRs, percentages), simple linear regression, and multiple linear regression to adjust for demographic confounders. |
|  |  | (b) Describe any methods used to examine subgroups and interactions | **8** | Subgroups (by sector, healthcare setting, and location) were evaluated as independent predictors in the adjusted regression model. |
|  |  | (c) Explain how missing data were addressed | **8, 9** | Complete-case analysis. Questionnaires were excluded *only* if an entire major evaluation section (like the complete perception or barrier scale) was left blank (n=33 excluded). |
|  |  | (d) Describe analytical methods taking account of sampling strategy | **5, 7, 8** | Convenience sampling with multi-channel recruitment was employed; no complex survey weights were applicable. |
|  |  | (e) Describe any sensitivity analyses | **N/A** | *Justification:* No clinical sensitivity analyses were planned or performed. |
| **RESULTS** |  |  |  |  |
| **Participants** | **13*** | (a) Report numbers of individuals at each stage of study—eg numbers potentially eligible, examined for eligibility, confirmed eligible, included in the study, completing follow-up, and analysed | **9** | "A total of 550 physicians were invited... 330 completed (60% response rate)... 33 excluded... leaving a final sample of 297..." |
|  |  | (b) Give reasons for non-participation at each stage | **9** | Severe clinical time constraints, busy schedules of clinicians, and electronic fatigue. |
|  |  | (c) Consider use of a flow diagram | **N/A** | *Justification:* Simple complete-case cohort attrition is fully and clearly reported textually in the first paragraph of the Results. |
| **Descriptive data** | **14*** | (a) Give characteristics of study participants (eg demographic, clinical, social) and information on exposures and potential confounders | **9, 10** | Fully reported in "Table 1. Sociodemographic characteristics of the participating physicians (n= 297)". |
|  |  | (b) Indicate number of participants with missing data for each variable of interest | **9, 10** | Missing data was handled at entry; Table 1 shows 100% complete variables for the n=297 analyzed cohort. |
|  |  | (c) State any follow-up time (e.g., average and total) | **N/A** | *Justification:* This is a cross-sectional survey; no longitudinal follow-up was performed. |
| **Outcome data** | **15*** | Report numbers of outcome events or summary measures | **9–12** | Outcome distributions are comprehensively summarized in Figure 1, Figure 2, Table 2, and Table 3. |
| **Main results** | **16** | (a) Give unadjusted estimates and, if applicable, confounder-adjusted estimates and their precision (eg, 95% confidence interval). Make clear which confounders were adjusted for and why they were included | **12, 13** | Both simple (unadjusted) and multiple (adjusted) linear regression results ($B$, 95% CI, and $p$-values) are reported in Table 4. |
|  |  | (b) Report category boundaries when continuous variables were categorized | **7, 10** | Boundaries for the Likert scales (1–5) and composite scores (8–40) are described; demographics are kept continuous as medians with IQRs. |
|  |  | (c) If relevant, consider translating estimates of relative risk into an absolute risk for a meaningful time period | **N/A** | *Justification:* Causal relative risk and absolute risk estimates are not applicable to cross-sectional association regression models. |
| **Other analyses** | **17** | Report other analyses done—eg analyses of subgroups and interactions, and sensitivity analyses | **12, 13** | Subgroup factors are analyzed as independent covariates in the multiple linear regression model (Table 4). |
| **DISCUSSION** |  |  |  |  |
| **Key results** | **18** | Summarise key results with reference to study objectives | **13–16** | Key findings summarized: high collaboration rates, positive clinical views, sector differences (lower views in private sector), and major organizational barriers. |
| **Limitations** | **19** | Discuss limitations of the study, taking into account sources of potential bias or imprecision. Discuss both direction and magnitude of any potential bias | **16, 17** | We explicitly discuss selection bias (moderate magnitude, upward/positive direction), scale validation parameters, setting ambiguity, and 5.66% margin of error. |
| **Interpretation** | **20** | Give a cautious overall interpretation of results considering objectives, limitations, multiplicity of analyses, results from similar studies, and other relevant evidence | **13–16** | Carefully contextualized in light of traditional role boundaries, Jordan's fee-for-service private model, public sector resource gaps, and international models (Slovenia/Italy). |
| **Generalisability** | **21** | Discuss the generalisability (external validity) of the study results | **17** | Discussed in "Strengths and Limitations" section, noting that convenience sampling limits absolute census generalizability and presents the study as an exploratory baseline. |
| **OTHER INFORMATION** |  |  |  |  |
| **Funding** | **22** | Give the source of funding and the role of the funders for the present study and, if applicable, for the original study on which the present article is based | **19** | "Funding: This research received no specific grant from any funding agency in the public, commercial, or not-for-profit sectors." |
